# Drug interaction between methotrexate and salazosulfapyridine in Japanese patients with rheumatoid arthritis

**DOI:** 10.1186/s40780-017-0073-z

**Published:** 2017-01-19

**Authors:** Morihiro Okada, Hiroshi Fujii, Yukio Suga, Satoshi Morito, Masae Okada, Jun Nishigami, Mitsuhiro Kawano, Tsutomu Shimada, Yoshimichi Sai

**Affiliations:** 1 Department of Pharmacy, Japan Community Healthcare Organization Kanazawa Hospital, Ha-15 Oki-machi, Kanazawa, 920-8610 Japan; 2 Department of Medicinal Informatics, Graduate School of Medical Sciences, Kanazawa University, 13-1 Takara-machi, Kanazawa, 920-8641 Japan; 3 Division of Rheumatology, Kanazawa University Graduate School of Medicine, 13-1 Takara-machi, Kanazawa, 920-8641 Japan; 4 Institute of Medical, Pharmaceutical and Health Science, Kanazawa University, 13-1 Takara-machi, Kanazawa, 920-8641 Japan; 5 Department of Pharmacy, Social Welfare Organization Saiseikai Kanazawa Hospital, 13-6 Akatsuti-machi, Kanazawa, 920-0353 Japan; 6 Department of Hospital Pharmacy, University Hospital, Kanazawa University, 13-1 Takara-machi, Kanazawa, 920-8641 Japan

**Keywords:** Rheumatoid arthritis, MTX, SASP, Polypharmacy

## Abstract

**Background:**

Methotrexate (MTX) and salazosulfapyridine (SASP) are disease-modifying drugs that are commonly used in the treatment of rheumatoid arthritis (RA), and combination therapy with MTX and SASP is recommended for RA patients who show an inadequate response to monotherapy with either drug. This study was designed to examine the interaction between the two drugs from the viewpoint of serum MTX concentration in Japanese RA patients, who were receiving combination therapy with relatively low doses of MTX and SASP.

**Methods:**

This is a 24-week open-label intervention study of stable RA patients (*n* = 10) with low disease activity. In these patients, who had received SASP/MTX combination therapy for at least 12 weeks, SASP was discontinued, and the patients received MTX monotherapy for the next 24 weeks. The primary outcome was change of serum MTX concentration at 12 weeks after discontinuation of SASP. Two disease activity markers, simplified disease activity index (SDAI) and disease activity score-C reactive protein (DAS28-CRP), were assessed as secondary outcomes at 24 weeks after discontinuation of SASP. We also monitored levels of matrix metalloproteinase-3 (MMP-3) and inflammatory cytokines. Patients were asked to complete a questionnaire after the study.

**Results:**

Serum MTX concentration in RA patients who discontinued SASP increased more than 2-fold within 4 weeks, and the higher level was maintained thereafter. No significant differences were detected in SDAI, DAS28-CRP, MMP-3 or inflammatory cytokines. Most participants reported no change in physical condition after withdrawal of SASP, and most preferred MTX monotherapy for future treatment.

**Conclusions:**

Withdrawal of SASP from patients receiving SASP/MTX caused a rapid, marked increase of serum MTX concentration, without any apparent change in disease parameters or side effects. Our results suggest that SASP can be discontinued without adverse effects in stable RA patients receiving combination therapy, at least among Japanese patients receiving relatively low doses of the two drugs.

**Trial registration:**

UMIN000024507. October 21, 2016 retrospectively registered.

## Background

Rheumatoid arthritis (RA) is a chronic, systemic, destructive joint disease. It occurs most frequently in middle-aged women, and about 0.5 to 1.0% of the population suffer from the disease in the industrialized world [1]. At onset, patients report morning stiffness and painful swelling of small joints. These symptoms eventually cause irreversible destruction of bone and cartilage [2], leading to severe disability in daily life. Therefore, early diagnosis and early intervention to prevent joint destruction are important.

The symptoms are caused by inflammatory cytokines, such as tumor necrosis factor-α (TNF-α) and interleukin-6 (IL-6). Methotrexate (MTX) and salazosulfapyridine (SASP) are considered as first-choice drugs to treat RA [3, 4]. MTX was developed as an anti-cancer drug in the 1940s, and was subsequently approved for RA in 1999 in Japan; it has a cytostatic effect due to its folic acid metabolism antagonistic activity, and also has an anti-inflammatory action by increasing production of adenosine. There is abundant evidence supporting the efficacy, economy, and tolerance of MTX in RA patients [5]. On the other hand, SASP has been used to treat RA since 1995 in Japan. It has antibacterial and anti-inflammatory activities, and is an immunomodulator [6]. SASP is often prescribed for RA patients who show low disease activity or who respond poorly to MTX, or who have encountered adverse events of MTX. Further, SASP/MTX combination therapy is often used for RA patients who do not show an adequate response to monotherapy, since it was found in a clinical trial that combination therapy with MTX and SASP is superior to either drug alone [7]. The American College of Rheumatology (ACR) and European League Against Rheumatism (EULAR) recommend combination therapy with disease-modifying anti-rheumatic drugs (DMARDs) in RA patients who have high disease activity and poor prognosis [3, 4]. However, a meta-analysis of combination therapy with MTX and SASP found no superiority over monotherapy with MTX [8].

MTX is absorbed through mainly via the proton-coupled folate transporter (PCFT) in the upper small intestine [9, 10]. In vitro transport kinetic analyses indicated that SASP is a potent inhibitor of PCFT-mediated cellular uptake of MTX [11], suggesting that the absorption rate of MTX would be reduced in patients receiving the combination therapy. However, it is not known whether this change in the pharmacokinetics of MTX also occurs in RA patients, and if so, whether it influences the efficacy of SASP/MTX combination therapy.

In this study, we investigated the drug interaction of MTX and SASP in RA patients, with the aim of establishing whether SASP discontinuation is a reasonable strategy in patients receiving the combined therapy. This information is important, because polypharmacy is undesirable on various grounds. For this purpose, we examined the effect of SASP withdrawal in stable RA patients who had received the combination therapy. We measured serum MTX concentration, disease activity markers and the levels of matrix metalloproteinase-3 (MMP-3) and inflammatory cytokines, as well as administering a questionnaire to patients.

## Methods

### Participant population

Outpatients fulfilling the ACR criteria for diagnosis of RA were recruited [12]. The main inclusion criteria were age ≥ 20 years, treatment with SASP/MTX combination therapy for at least 12 weeks before entry into this study, and low disease activity, defined as simplified disease activity index (SDAI) ≤ 11 [13]. Thirteen patients who met these criteria agreed to participate in this study, but 3 patients who showed poor compliance were eliminated, leaving 10 patients (5 men, 5 women). The characteristics of participants at week 0 (discontinuation of SASP) are summarized in Table 1, i.e., sex, age, duration of RA, serum creatinine (Cr), blood urea nitrogen (BUN), aspartate aminotransferase (AST), alanine aminotransferase (ALT), mean corpuscular hemoglobin (MCV), C-reactive protein (CRP), SDAI, disease activity score-CRP (DAS28-CRP), MMP-3, dosage of MTX, dosage of SASP, dosage of prednisolone (PSL), and number of drugs being taken.

**Table 1:** Baseline clinical characteristics of participants

Items	Participants
No. of patients (male/female)	10 (5/5)
Age (years)	64.1 ± 10.6
Duration of RA (years)	8.4 ± 11.0
Cr (mg/dL)	0.66 ± 0.12
BUN (mg/dL)	15.6 ± 4.9
AST (IU/L)	20.5 ± 3.3
ALT (IU/L)	23.3 ± 11.6
MCV (fL)	97.8 ± 2.5
CRP (mg/dL)	0.2 ± 0.2
SDAI	5.4 ± 4.0
DAS28-CRP	2.1 ± 0.8
MMP-3 (ng/mL)	130.2 ± 88.6
MTX (mg/week)	7.0 ± 3.0
MTX dosage at first dose each week	
4 mg (No. of patients)	5
2 mg (No. of patients)	5
SASP (mg/day)	1000 ± 0
PSL (mg/day)	2.7 ± 2.7
No. of drugs being taken	6.0 ± 2.1

*Cr* Serum creatinine, *BUN* blood urea nitrogen, *AST* aspartate aminotransferase, *ALT* alanine aminotransferase, *MCV* mean corpuscular hemoglobin, *CRP* C-reactive protein, *SDAI* simplified disease activity index, *DAS28-CRP* disease activity score-C reactive protein, *MMP-3* matrix metalloproteinase-3, *MTX* methotrexate, *SASP* salazosulfapyridine and *PSL* prednisolone. Data are mean ± SD

### Study design

This prospective multicenter trial of 24 weeks duration was conducted in three hospitals (Kanazawa University Hospital, Social Welfare Organization Saiseikai Kanazawa Hospital, and Japan Community Healthcare Organization Kanazawa Hospital). The study was approved by the ethics committee of Kanazawa University, the ethics committee of Social Welfare Organization Saiseikai Kanazawa Hospital and the research ethics committee of Japan Community Healthcare Organization Kanazawa Hospital (approval number, 1228–2, 2012–002 and 11-01-02, respectively), and informed consent was obtained from all patients before initiation of the study. This study was conducted in accordance with the Declaration of Helsinki and Good Clinical Practice Guidelines.

### Treatment and observation

Serum MTX concentration was set as the primary outcome. MTX was administered orally twice or three times per week (total dosage: 4.0 - 12.0 mg per week). SASP was administered orally twice a day (total dosage: 1,000 mg per day). Blood samples were taken 2 h after the first administration of MTX in any given week, because the time-to-maximum serum MTX concentration was reported to be 2.0 ± 0.8 h in the case of oral administration [14]. At baseline (week 0), participants stopped taking SASP, and continued MTX monotherapy, and blood samples were taken 4, 8 and 12 weeks later to measure serum MTX. As secondary outcomes, we measured SDAI and DAS28-CRP at baseline (week 0) and at 4, 8, 12 and 24 weeks to evaluate disease activity, and MMP-3 at baseline (week 0) and at 12 and 24 weeks to estimate the cartilage destruction [15]. We also measured TNF-α and IL-6 in 4 participants at baseline (week 0) and at 4, 8 and 12 weeks to examine immunological changes. We monitored adverse events such as interstitial pneumonia, myelosuppression and infection through the period of observation. MTX dosage and the regimen of other drugs were kept unchanged during this study. Adherence was checked by interviewing the participants and by pill counting at each hospital visit. The questionnaire was completed at the end of the study.

### Serum MTX concentration assay

Serum was obtained by centrifugation of whole blood at 2,500 rpm for 5 min. The serum MTX concentration was measured by fluorescence polarization immunoassay with a TDxFLx analyzer (Abbot Lab., IL., USA).

### Evaluation of disease activity markers

We monitored SDAI and DAS28-CRP as markers of disease activity. SDAI was recommended as a standard marker by ACR and EULAR in 2011 [16]. SDAI was calculated from the number of swollen joints among 28 joints (SJ), tender joints among 28 joints (TJ), the patient’s global assessment of pain on a 10-cm visual analog scale (PG10), the physician’s global assessment of disease activity on a 10-cm visual analog scale (MDG10) and CRP, according to the following formula [13]:

$$ \mathrm{SDAI}=\mathrm{S}\mathrm{J}+\mathrm{T}\mathrm{J}+\mathrm{P}\mathrm{G}10+\mathrm{M}\mathrm{D}\mathrm{G}10+\mathrm{C}\mathrm{R}\mathrm{P} $$

SDAI scores were evaluated as follows: ≤3.3, remission; >3.3 and ≤11, low disease activity; >11 and ≤26, moderate disease activity; >26, high disease activity [17].

DAS28-CRP has been developed to discriminate between high and low disease activity as indicated by clinical decisions of rheumatologists since 20 year ago [18]. It was calculated from the number of SJ, TJ, CRP and the patient’s global assessment of pain on a 100-mm visual analog scale (PG100), according to the following formula [19]:

$$ \mathrm{D}\mathrm{A}\mathrm{S}28\hbox{-} \mathrm{C}\mathrm{R}\mathrm{P}=0.56\times \sqrt{TJ}\kern0.5em +0.28\times \sqrt{SJ}+0.36\times \ln \left(\mathrm{C}\mathrm{R}\mathrm{P}\times 10+1\right)+0.014\times \mathrm{P}\mathrm{G}100+0.96 $$

DAS28-CRP scores were evaluated as follows: ≤2.3, remission; >2.3 and ≤2.7, low disease activity; >2.7 and ≤4.1, moderate disease activity; >4.1, high disease activity [17].

### Assay of serum MMP-3, TNF-α and IL-6

Serum was obtained by centrifugation of whole blood at 2,500 rpm for 5 min. MMP-3 was measured by means of latex agglutination turbidimetry (BML, Inc., Tokyo, Japan). Serum levels of TNF-α and IL-6 were measured in 4 participants with ELISA, according to the manufacturer’s protocol (R&D Systems, Inc., MN., USA).

### Subjective evaluation by participants

We asked participants to complete a short questionnaire consisting of three questions at the end of the study. The first question is “Which was better for your physical condition, the combination therapy or the monotherapy”. The second question is “Which treatment would you choose to receive in the future, the combination therapy or the monotherapy”. The third question is “Please give the reasons for your choice in question 2”.

### Statistical analysis

All data were analyzed using IBM SPSS statistical software version 18 for Windows (SPSS Japan Inc., Tokyo, Japan). One-way repeated measures ANOVA with post hoc Tukey’s test was used for comparisons of numerical values. Data were expressed as the mean ± standard deviation. *P* values under 5% were considered to be significant.

## Results

### Changes of serum MTX concentration

**Fig. 1:**
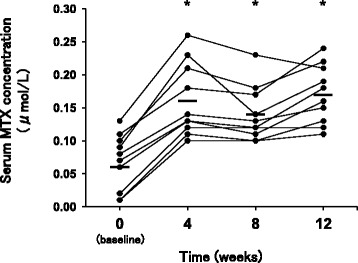
Changes of serum MTX concentration. The figure showed individual serum MTX concentrations at the conclusion of SASP/MTX combination therapy (baseline, 0 weeks) and at 4, 8 and 12 weeks after withdrawal of SASP. Horizontal bars represent the mean serum concentration for all participants (*n* = 10). Comparisons were made by one-way repeated measures ANOVA with post hoc Tukey’s test. ^*^ *p* < 0.005, significantly different from baseline

As shown in Fig. 1, participants in the study had significant higher serum MTX concentrations in the MTX monotherapy phase than in the SASP/MTX combination phase. The value of MTX concentration at the end of the combination therapy phase (baseline, week 0) was 0.07 ± 0.04 μmol/L. The corresponding values after withdrawal of SASP were: 4 weeks, 0.16 ± 0.06 μmol/L; 8 weeks, 0.14 ± 0.04 μmol/L; 12 weeks, 0.17 ± 0.04 μmol/L; *p* < 0.005, respectively).

### Changes of disease activity

**Fig. 2:**
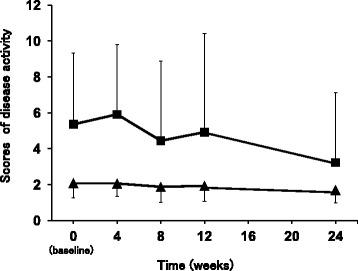
Changes of participants’ disease activity scores. The figure showed the scores of SDAI and DAS28-CRP at the conclusion of SASP/MTX combination therapy (baseline, 0 weeks), and at 4, 8, 12 and 24 weeks after withdrawal of SASP. The squares indicated SDAI (■). The triangles indicated DAS28-CRP (▲). Data are mean ± SD (*bars*) for all assigned participants (*n* = 10). Comparisons were made by one-way repeated measures ANOVA with post hoc Tukey’s test

No statistically significant differences were detected in SDAI (Fig. 2), although there was a tendency of improvement at 24 weeks. The baseline value at week 0 was 5.4 ± 4.0 points. Subsequent values were: 4 weeks, 5.9 ± 3.9 points; 8 weeks, 4.5 ± 4.4 points; 12 weeks, 4.9 ± 5.5 points; 24 weeks, 3.2 ± 3.9 points. DAS28-CRP showed no significant change during the study. The baseline value at the beginning of the study (week 0) was 2.1 ± 0.8 points. Subsequent values were: 4 weeks, 2.1 ± 0.7 points; 8 weeks, 1.9 ± 0.9 points; 12 weeks, 1.9 ± 0.9 points; 24 weeks, 1.7 ± 0.7 points).

### Changes of serum MMP-3 and cytokine levels

**Fig. 3:**
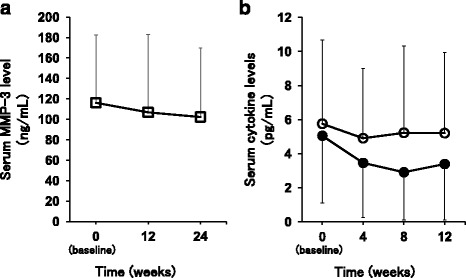
Changes of serum MMP-3 and cytokine levels. **a** The left figure shows serum MMP-3 (□) levels (mean ± SD (*bars*), *n* = 10) at the conclusion of SASP/MTX combination therapy (baseline, 0 weeks), and at 12 and 24 weeks after withdrawal of SASP. **b** The right figure shows serum TNF-α (●) and the serum IL-6 (○) levels (mean ± SD (bars), *n* = 4) at the conclusion of SASP/MTX combination therapy (baseline, 0 weeks), and at 4, 8 and 12 weeks after withdrawal of SASP. Comparisons were made by one-way repeated measures ANOVA with post hoc Tukey’s test

As shown in Fig. 3a, MMP-3 showed no significant change during the 24-week study (baseline, week 0, 116.1 ± 66.3 ng/mL; 12 weeks, 106.9 ± 76.1 ng/mL; 24 weeks, 102.3 ± 67.6 ng/mL). As shown in Fig. 3b, there was no statistically significant change of TNF-α or IL-6 after withdrawal of SASP. The values of TNF-α and IL-6 at baseline (week 0) were 5.1 ± 7.4 pg/mL and 5.6 ± 4.9 pg/mL, and those after discontinuation of SASP were 3.5 ± 4.7 pg/mL and 4.9 ± 4.1 pg/mL at 4 weeks, 2.9 ± 3.1 pg/mL and 5.2 ± 5.2 pg/mL at 8 weeks, 3.4 ± 4.3 pg/mL and 5.2 ± 4.7 pg/mL at 12 weeks, respectively.

### Subjective evaluation of participants

The questionnaire results are shown in Table 2. Seven out of 10 participants reported no change in physical condition after withdrawal of SASP, while the remaining 3 felt better after the withdrawal. Eight out of 10 said they would prefer MTX monotherapy in future. Interestingly, participants who favored monotherapy in the future said that a major reason for their choice was to reduce the number of drugs they were taking, i.e., they saw polypharmacy as undesirable.

**Table 2:** Questionnaire: subjective evaluation by participants

Questionnaire	No. of participants
Question 1: Which was better for your physical condition, the combination therapy or the monotherapy? (*n* = 10)	
Combination therapy	0
Monotherapy	3
I found no difference	7
Question 2: Which treatment would you choose to receive in the future, the combination therapy or the monotherapy? (*n* = 10)	
Combination therapy	0
Monotherapy	8
Follow primary physician’s advice.	2
Question 3: Please give the reasons for your choice in question 2. (*n* = 8, multiple answers allowed)	
I want to reduce the number of drugs I am taking	6
I felt better with monotherapy	3
I am not worried about side effects	2
The cost of drugs is important to me	1

### Occurrence of serious adverse events

None of the participants experienced serious adverse events in this study. Values of Cr, BUN, AST, ALT and MCV were unchanged during the study (Cr: 0.66 ± 0.12 mg/dL at baseline (week 0) to 0.67 ± 0.14 mg/dL at 24 weeks, BUN: 15.6 ± 4.9 mg/dL at baseline to 13.7 ± 3.3 mg/dL at 24 weeks, AST: 20.5 ± 3.3 IU/L at baseline to 20.2 ± 4.1 IU/L at 24 weeks, ALT: 23.3 ± 11.6 IU/L at baseline to 22.3 ± 14.1 IU/L at 24 weeks, MCV: 97.8 ± 2.5 fL at baseline to 96.0 ± 4.0 fL at 24 weeks).

## Discussion

We found that the serum MTX concentration in RA patients who were taking SASP/MTX combination therapy increased more than 2-fold when SASP was discontinued, and the increase was maintained thereafter. It was reported that improvement of inflammatory markers in RA patients is correlated with the time for which the serum MTX concentration is over 0.02 μM [20]. In this study, we measured only the maximum serum MTX concentration since it was not feasible to carry out frequent blood samplings in outpatients. Three out of 10 participants receiving the combination therapy had Cmax values of MTX lower than 0.02 μM. But, after withdrawal of SASP, all participants showed a higher maximum serum MTX concentration than 0.02 μM. Furthermore, the MTX concentration at baseline in this study was lower than the average serum MTX concentration in Japanese RA patients who received weekly pulse low-dose MTX therapy in a prior study [14], supporting the idea that coadministration of SASP tends to decrease MTX concentration.

It is noteworthy that SDAI and TNF-α tended to decrease after SASP withdrawal, and no patient showed exacerbation of RA status (Figs. 2 and 3). These results suggest that the increased serum MTX concentration following SASP withdrawal at least compensated for the loss of the efficacy of the SASP. These findings appear to be inconsistent with the ACR and EULAR recommendations for combination therapy with MTX and SASP [3, 4]. However, the apparent contradiction may be explained by differences in the dosages of MTX and SASP. Our participants received an average MTX dosage of 7.0 mg/week, compared with 12.5 mg/week in Capell et al.’s clinical trial in the UK [7]. Similarly, the average SASP dosage in our participants was 1,000 mg/day, compared with 2,500 mg/day in Capell et al.’s clinical trial [7]. In other words, Japanese RA patients take smaller dosages of MTX and SASP than those recommended in ACR and EULAR. It seems likely that these relatively low MTX and SASP dosages are unsuitable for combination therapy for RA patients.

The reason for the increased serum MTX concentration following SASP withdrawal may be that SASP is a potent inhibitor of PCFT, which plays an essential role in MTX absorption from the upper small intestine. Indeed, the influx of MTX is inhibited by SASP in human alveolar epithelial cell line A549 expressing PCFT mRNA [10]. It was also reported that the IC_50_ of SASP for MTX uptake in HEK293 cells stably expressing human PCFT was 60.4 μM [11]. In the clinical situation, the nominal SASP concentration might be 5 mM or higher in the small intestine when 500 mg of SASP is taken with 200 mL of water, and so the concentration of SASP should be more than sufficient to inhibit the absorption of MTX via PCFT. We consider that the recovery of PCFT function plays a key role in the recovery of serum MTX concentration after SASP withdrawal from SASP/MTX combination therapy. A possible countermeasure to overcome the MTX-suppressing effect of combination therapy might be to take SASP only in the during the intervening periods which not to take MTX under weekly pulse low-dose MTX therapy, since orally administered MTX was rapidly and almost completely absorbed [21].

The results of the questionnaire indicated that most participants preferred MTX monotherapy. Thus, on the basis of both objective and subjective evaluations, it would be reasonable to withdraw SASP in stable RA patients receiving SASP/MTX combination therapy. In this study, the resulting increase of serum MTX concentration did not cause severe adverse events. However, care is necessary in the case of SASP withdrawal, because MTX-associated adverse events such as bone marrow suppression and liver injury are MTX dose-dependent. Further study will be needed to identify the optimum treatment in RA patients undergoing DMARDs therapy.

Recently, polypharmacy has become a significant social problem in our aging society [22]. Schuler et al.’s reported that the average number of prescriptions in 543 patients who entered hospital in Austria was 7.5 ± 3.8. They identified possible drug-drug interaction in 65.8% and adverse events in 17.8% of the patients [23]. In another study, the incidence of adverse events was 6.5% when patients took 1–3 drugs, and 13.1% when they took 6–7 drugs [24]. It has also been demonstrated in a clinical trial that the incidence of nausea was increased in RA patients taking SASP/MTX combination therapy compared with MTX monotherapy [25]. Polypharmacy is a particularly important problem for RA patients, who often take large numbers of drugs, such as biological agents, DMARDs, prednisolone (PSL), non-steroidal anti-inflammatory drugs, stomach drugs and anti-osteoporosis drugs. Among our stable RA patients, the average number of drugs was 6.0 ± 2.1. Thus, if we can reduce polypharmacy by replacing SASP/MTX combination therapy with MTX monotherapy based on the results of this study, there could be a significant benefit for RA patients.

The present study has several important limitations. First, the number of cases was small. Secondly, we used an open label design, which might have introduced subjective bias. Thirdly, we did not examine changes of methotrexate-polyglutamates, which accumulate in cells during MTX administration, though a relationship between disease activity of RA and methotrexate-polyglutamates concentration was recently reported [26]. Nevertheless, we believe the results of this small-scale study warrant a more detailed re-examination of the relative merits of SASP/MTX combination therapy and MTX monotherapy.

## Conclusions

The results of this small-scale open-label clinical trial indicate that the MTX concentration increased markedly upon discontinuation of SASP, with no apparent change of efficacy or side effects. Therefore, we suggest that it is reasonable to withdraw SASP in stable RA patients receiving SASP/MTX combination therapy, at least in Japanese RA patients receiving relatively low doses of the two agents. This would have the advantage of reducing the potential adverse effects of polypharmacy.

## Abbreviations

ACR: American College of Rheumatology
DAS-28CRP: Disease activity score-C reactive protein
DMARDs: Disease-modifying anti-rheumatic drugs
EULAR: European League Against Rheumatism
IL-6: Interleukin-6
MCV: Mean corpuscular hemoglobin
MMP-3: Matrix metalloproteinase-3
MTX: Methotrexate
PCFT: Proton-coupled folate transporter
PSL: Prednisolone
RA: Rheumatoid arthritis
SASP: Salazosulfapyridine
SDAI: Simplified disease activity index
TNF-α: Tumor necrosis factor
